# Functional Homologous Recombination (HR) Screening Shows the Majority of *BRCA1/2*-Mutant Breast and Ovarian Cancer Cell Lines Are HR-Proficient

**DOI:** 10.3390/cancers16040741

**Published:** 2024-02-10

**Authors:** Titia G. Meijer, John W. M. Martens, Wendy J. C. Prager-van der Smissen, Nicole S. Verkaik, Corine M. Beaufort, Stanley van Herk, Teresa Robert-Finestra, Remco M. Hoogenboezem, Kirsten Ruigrok-Ritstier, Maarten W. Paul, Joost Gribnau, Eric M. J. Bindels, Roland Kanaar, Agnes Jager, Dik C. van Gent, Antoinette Hollestelle

**Affiliations:** 1 Department of Molecular Genetics, Erasmus University Medical Center, 3015 GD Rotterdam, The Netherlands; 2 Department of Pathology, Erasmus University Medical Center, 3015 GD Rotterdam, The Netherlands; 3 Oncode Institute, 3521 AL Utrecht, The Netherlands; 4 Department of Medical Oncology, Erasmus MC Cancer Institute, Erasmus University Medical Center, 3015 GD Rotterdam, The Netherlands; 5 Department of Hematology, Erasmus MC Cancer Institute, Erasmus University Medical Center, 3015 GD Rotterdam, The Netherlands; 6 Department of Developmental Biology, Erasmus University Medical Center, 3015 GD Rotterdam, The Netherlands

**Keywords:** homologous recombination repair, functional status, breast cancer, ovarian cancer, cell lines, BRCA1/2, mutation status, mutational signature

## Abstract

**Simple Summary:**

Cancer cells from patients who carry a disease-causing *BRCA1* or *BRCA2* mutation are incapable of repairing double-strand DNA breaks via the homologous recombination (HR) repair pathway. As a consequence, these patients respond to platinum-based chemotherapy and Poly-[ADP-Ribose]-Polymerase inhibitors (PARPi). However, using a functional read-out for HR, we show that not all HR-deficient cell lines carry a *BRCA1/2* mutation. Moreover, the majority of *BRCA1/2*-mutant cell lines are HR proficient, highlighting the importance of measuring the functional HR status. For almost all of the *BRCA1/2*-mutant HR proficient cell lines, we could explain the discrepant HR status. The observed reversion mechanisms predominantly acted on restoring the *BRCA1* or *BRCA2* mutation directly. Taken together, our results indicate that only determining *BRCA1/2* mutation status may not suffice for selecting patients for platinum-based chemotherapy or PARPi. Moreover, for experimental approaches cell line functionality of HR should be assessed beforehand.

**Abstract:**

Tumors with a pathogenic *BRCA1/2* mutation are homologous recombination (HR)-deficient (HRD) and consequently sensitive to platinum-based chemotherapy and Poly-[ADP-Ribose]-Polymerase inhibitors (PARPi). We hypothesized that functional HR status better reflects real-time HR status than *BRCA1/2* mutation status. Therefore, we determined the functional HR status of 53 breast cancer (BC) and 38 ovarian cancer (OC) cell lines by measuring the formation of RAD51 foci after irradiation. Discrepancies between functional HR and *BRCA1/2* mutation status were investigated using exome sequencing, methylation and gene expression data from 50 HR-related genes. A pathogenic *BRCA1/2* mutation was found in 10/53 (18.9%) of BC and 7/38 (18.4%) of OC cell lines. Among *BRCA1/2*-mutant cell lines, 14/17 (82.4%) were HR-proficient (HRP), while 1/74 (1.4%) wild-type cell lines was HRD. For most (80%) cell lines, we explained the discrepancy between functional HR and *BRCA1/2* mutation status. Importantly, 12/14 (85.7%) *BRCA1/2*-mutant HRP cell lines were explained by mechanisms directly acting on BRCA1/2. Finally, functional HR status was strongly associated with COSMIC single base substitution signature 3, but not *BRCA1/2* mutation status. Thus, the majority of *BRCA1/2*-mutant cell lines do not represent a suitable model for HRD. Moreover, exclusively determining *BRCA1/2* mutation status may not suffice for platinum-based chemotherapy or PARPi patient selection.

## 1. Introduction

Germline *BRCA1/2* mutations underlie tumorigenesis in approximately 5–15% of all ovarian cancer (OC), 3% of all breast cancer (BC) and in 10–20% of all triple negative BC cases [1,2,3]. The BRCA1/2 proteins play a pivotal role in the error-free repair of DNA double-strand breaks (DSB) via the homologous recombination (HR) pathway. Therefore, tumors with a pathogenic *BRCA1/2* mutation, in general, are HR deficient (HRD) and thus highly sensitive to Poly-[ADP-Ribose]-Polymerase inhibitors (PARPi) and DSB-inducing chemotherapies, such as platinum-based compounds [4,5,6,7]. However, HRD has also been observed in tumors without a germline *BRCA1/2* mutation. According to a variety of HRD tests, approximately 20% of primary BC and up to 50% of OC are HRD and could thus also benefit from PARPi [8,9,10,11].

Unfortunately, resistance to PARPi has emerged as a new challenge in the clinic. Various resistance mechanisms exist and they can be classified into four categories: increased drug efflux, decreased PARP trapping, restoration of HR and stabilization of stalled replication forks [12,13]. Resistance to PARPi is also associated with resistance to platinum-containing compounds, indicating overlapping mechanisms [14]. A number of resistance mechanisms have been identified in pre-clinical genetic screens or in studies with patient-derived xenografts (PDXs) [15,16,17]. However, clinical evidence exists only for reversion mutations in the *BRCA1/2* genes, causing restoration of the BRCA1/2 gene function [18,19].

In light of all this, accurately identifying patients who respond to PARPi treatment is imperative. Since not all patients with an HRD phenotype carry a mutation in either *BRCA1* or *BRCA2*, only screening these two genes will be insufficient for accurate stratification. In this respect, HRD causes specific mutational scars in the genome that can be detected via massive parallel sequencing, such as single base substitution (SBS) signatures 3 and 8 (SBS3 and SBS8), small insertion/deletion (ID) signature (ID6) and structural variant (SV) signatures 3 and 5 (SV3 for BRCA1-type cancers and SV5 for BRCA2-type cancers) [20,21,22]. However, these mutational scars will remain part of the genome despite evolving drug resistance due to HR restoration. Therefore, measuring HRD status via a functional assay is necessary for real-time HR status assessment and consequently monitoring drug resistance before and during treatment.

For pre-clinical studies, human cancer cell lines are the most commonly used model system. Although the caveats of cell line studies are well-known, such as cross-contamination and genetic drift, a well-described collection of cell lines is a valuable source of biological material for studying molecular mechanisms and to perform drug screening [23]. With this in mind, we decided to determine the functional HR status in our large panel of BC (*n* = 53) and OC cell lines (*n* = 38) using our in-house developed RECAP assay [24,25,26].

We hypothesized that the functional HR status of cell lines would better reflect the real-time HR status of the cells at the time of testing than the original germline *BRCA1/2* mutation status. Therefore, we performed a comprehensive characterization of our large collection of BC and OC cell lines to study the discrepancies between functional HR and *BRCA1/2* mutation status as well as SBS signature 3. To study discrepancies in functional HRD and BRCA1/2 mutation status, we investigated exome sequencing, gene expression and methylation data for a panel of 50 known HRD genes.

## 2. Materials and Methods

### 2.1. BC and OC Cell Lines

**Figure 1:**
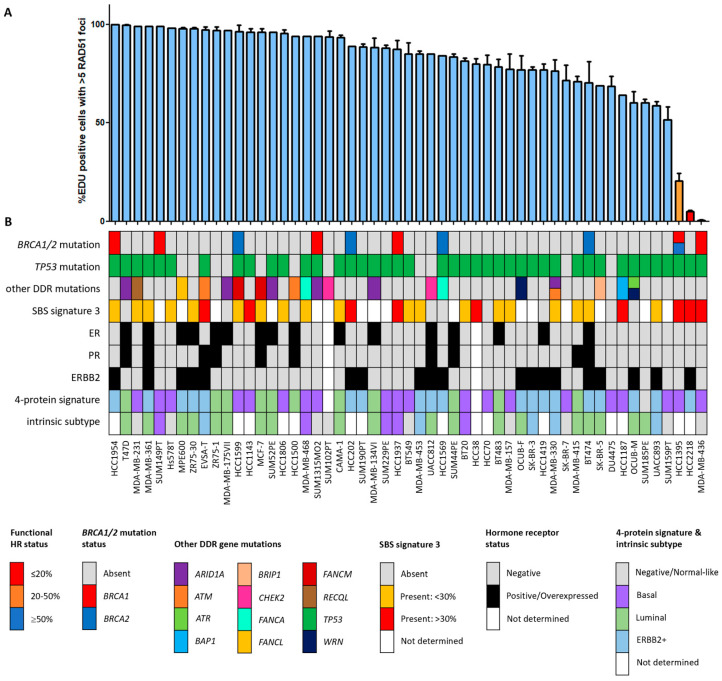
Functional HRD status of 53 breast cancer cell lines. (**A**) Quantification of the fraction of EdU-positive cells with ≥5 RAD51 foci. Each bar represents the mean value of three separate quantifications of the same cell line. Error bars indicate standard error of the mean (SEM). (**B**) Overview of the molecular and cellular characteristics of the cell lines. HR, homologous recombination; DDR, DNA damage response; SBS, single base substitution.

**Figure 2:**
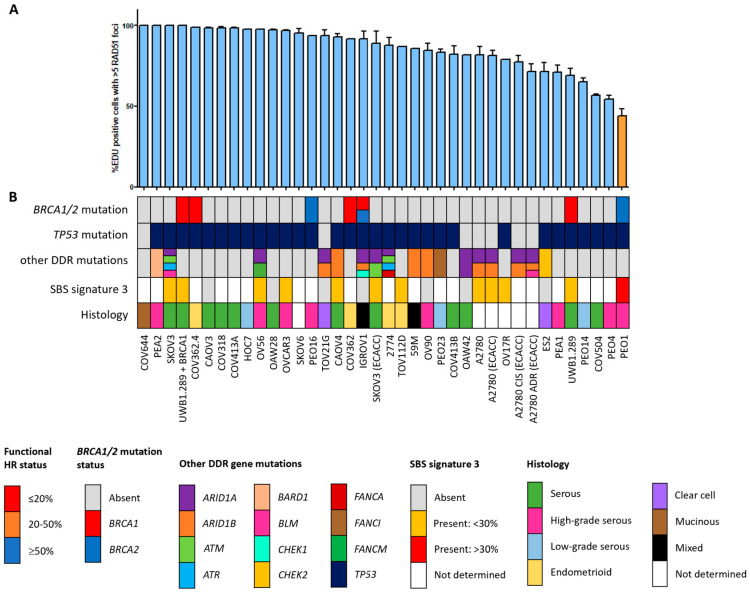
Functional HRD status of 38 ovarian cancer cell lines. (**A**) Quantification of the fraction of EdU-positive cells with ≥5 RAD51 foci. Each bar represents the mean value of three separate quantifications of the same cell line. Error bars indicate SEM. (**B**) Overview of the molecular and cellular characteristics of the cell lines. HR, homologous recombination; DDR, DNA damage response; SBS, single base substitution.

Fifty-three BC and thirty-eight OC cell lines (listed in Figure 1 and Figure 2 and Tables S1, S2 and S4) with confirmed identity and known origin [25,27,28] were cultured in RPMI 1640 medium (Thermo Fisher Scientific, Waltham, MA, USA) containing 10% FBS (Lonza, Walkersville, MD, USA) and penicillin/steptomycin (Thermo Fisher Scientific) at 37 °C and 5% CO_2_.

A2780 and A2780 (ECACC) as well as SKOV3 and SKOV3 (ECACC) are the same cell line, except they have a different origin (academic versus commercial, respectively). A2780 ADR and A2780 CIS are derived from the commercial line and were challenged once a month with 100 nM doxorubicin and 1 mM cisplatin, respectively. COV362.4 is a subclone of COV362 established by agar cloning. OCUB-F is a subclone of OCUB-M and was established by passaging non-adherent cells only. UWB1.289 is a BRCA1-null cell line, while UWB1.289+BRCA1 is an isogenic line stably transfected with a pcDNA3 plasmid containing a wild-type BRCA1 cDNA. The culture medium of UWB1.289+BRCA1 was supplemented with 200 µg/mL geneticin (G418 Sulfate; Thermo Fisher Scientific) to maintain selection pressure for transfected cells.

COV413A and COV413B, PEA1 and PEA2, PEO1 and PEO4, as well as PEO14 and PEO23 were cell line pairs derived from the same patient at different time points. COV413A and COV413B were derived from a colon and bladder dome metastasis, respectively. PEA1 was established from the treatment-naïve primary tumor, whereas PEA2 was established from the same tumor after developing platinum resistance 6 months after treatment. PEO1 was derived from the patient’s first platinum-sensitive relapse and PEO4 from a platinum-resistant relapse. PEO4 has a secondary BRCA2 reversion mutation that restores the original BRCA2 mutation that was present in PEO1. PEO14 was derived from the original treatment-naïve tumor, whereas PEO23 was derived from the platinum-resistant relapse.

### 2.2. Whole Exome Sequencing

Genomic DNA from cell lines was isolated using the NucleoSpin Tissue kit (Macherey-Nagel, Düren, Germany) according to manufacturer’s recommendations. The quality and quantity of the DNA was verified using NanoDrop 2000 measurement (A260/A280 = 1.8–2.0), gel electrophoresis (DNA length ≥ 10 kb) and Qubit measurement (Thermo Fisher Scientific). For each cell line, 250 ng of genomic DNA was used for library preparation using the Lotus™ DNA Library Prep Kit (Integrated DNA Technologies (IDT), Coralville, IA, USA). Enzymatic fragmentation and adapter ligation were performed according to the manufacturer’s recommendations, while the final library amplification step was performed using KAPA HiFi polymerase instead of the polymerase supplied with the kit (Roche, Basel, Switzerland). DNA libraries were verified on a Bioanalyzer 2100 (Agilent Technologies, Santa Clara, CA, USA) using the DNA1000 chip and pooled per 6 samples (500 ng per cell line totaling to a pooled library of 3 µg). Subsequent exome capture was performed using the xGEN Exome Research Panel v1.0 (IDT) using the manufacturer’s recommendations. Post-capture libraries were verified using the BioAnalyzer 2100 (Agilent) on a high-sensitivity DNA chip. Libraries were quantified using the NEBNext Library Quant kit (New England Biolabs, Ipswich, MA, USA), normalized to 2 nM and pooled.

Pooled libraries were 100 bp paired-end sequenced on a NovaSeq6000 (Illumina, San Diego, CA, USA) generating ~115M reads per cell line (obtaining an average of 30× per cell line). Reads were trimmed using Cutadapt v2.10 (https://doi.org/10.14806/ej.17.1.200, accessed on 4 June 2021) and aligned against hg19 using Burrows Wheeler Aligner v0.7.17 [29] with default settings. Samtools v1.12 [30] was used to sort and mark duplicate reads and Picard tools v2.17.10 (http://broadinstitute.github.io/picard/, accessed on 4 June 2021) for collecting quality metrics. Variants were called using Strelka v2.9.10 [31] in germline mode and the resulting VCFs were annotated using Annovar v10.2019 [32] and annotate_bam_statistics (https://github.com/RemcoHoogenboezem/annotate_bam_statistics_sc, accessed on 4 June 2021). Non-frequent (in <3 of 91 cell lines) recurring variants affecting 50 HR-related genes (listed in Table S3) identified in the 53 breast and 38 ovarian cancer cell lines are summarized in Tables S1 and S2, respectively.

### 2.3. RAD51 Immunofluorescence

Cells were seeded on coverslips one day prior to irradiation with 5 Gy using an X-ray machine (RS320, XStrahl Medical & Life sciences, Surrey, UK). For suspension cell lines, cells were irradiated in suspension before preparation of cytospins. One hour after irradiation, 3 μg/mL 5-Ethynyl deoxyuridine (EdU; Thermo Fisher Scientific) was added to label cells in S phase. Two hours after irradiation, cells were subjected to pre-extraction, fixation and RAD51 immunofluorescence as described previously [33]. Additionally, the presence of 53BP1/γH2AX foci was used as a positive control for the presence of DNA DSBs. Primary antibodies used were rabbit anti-RAD51 (1:10,000, [34]), rabbit anti-53BP1 (1:1000, NB100-904, Novus Biologicals, Centennial, CO, USA) and mouse anti-γH2AX (1:500, JBW301, Merck Millipore, Darmstadt, Germany). Secondary antibodies were Alexa Fluor^®^ 594 goat anti-mouse (1:1000) and Alexa Fluor^®^ 488 goat anti-rabbit (1:1000) (Thermo Fisher Scientific). Image acquisition was performed on a Leica TCS SP5 confocal microscope, using the 63× oil immersion objective. RAD51 foci in EdU positive cells were quantified manually. Per quantification, at least 30 EdU positive nuclei were counted and three independent RAD51 quantifications were performed per cell line. Cell lines were regarded as HRP if ≥50% and HRD if ≤20% of EdU positive cells contained ≥5 RAD51 foci. Cell lines were HR intermediate (HRI) if the percentage of EdU positive cells showing ≥5 RAD51 foci was between 20 and 50%. This classification was based on previous experiments in tissue slices [26].

### 2.4. BRCA1/2 Western Blotting

Cells were scraped and lysed in Laemmli sample buffer (2% SDS, 10% Glycerol and 60mM Tris pH 6.8), heated to 95 °C for 5 min and protein concentration was measured using the Lowry protein assay [35]. Size separation of proteins was achieved on a 3–8% Tris-Acetate gel (Novex, Thermo Fisher Scientific) and proteins were transferred to a PVDF membrane by wet blotting at 300 mA for two hours at 4 °C in transfer buffer (0.4 M Glycine, 5 mM Tris, 20% Methanol). Next, the membrane was blocked in 3% dry skimmed milk in PBS with 0.05% Tween-20 for at least one hour. Primary antibodies against BRCA2 (1:1000, OP95, Calbiochem, San Diego, CA, USA), BRCA1 (1:500, OP92, Merck Millipore), PARP1 (1:5,000, C2-10, Enzo Life Sciences, Farmingdale, NY, USA) and α-tubulin (1:10,000, T5168, Sigma-Aldrich, Saint Louis, MO, USA) were diluted in blocking buffer and incubated overnight at 4 °C. The secondary antibody, HRP-conjugated sheep anti-mouse IgG (1:2000, Jackson ImmunoResearch, Suffolk, UK), was incubated for 2 h at room temperature. Then, ECL substrate was added and the signal was detected on the Alliance 4.7 (Uvitec Cambridge Cleaver Scientific, Warwickshire, UK). The bands were quantified using the ‘Analyze Gels’ function in Image J (version 1.50i) and equal protein loading was verified by the α-tubulin loading control.

### 2.5. DNA Methylation, Gene Expression and Mutational Signature Data

Genome-wide methylation profiles were generated previously for BC cell lines (*n* = 53) using the recently developed MeD-seq assay [36]. Data from individual *LpnPI* sites were summarized per CpG island, for which annotations were downloaded from Ensembl (https://www.ensembl.org, accessed on 11 November 2019). Data were normalized to the total read count per library and square root transformed. All individual CpG islands that were (partly) located within a window ranging from 1000 bp upstream to 1000 bp downstream of the transcription start site of selected genes were selected for further analysis.

Gene expression data for part of the BC and OC cell lines (*n* = 69) were generated previously (GSE9385 and GSE53418) and measured using the Affymetrix GeneChip Human Exon 1.0 ST Array [25,37]. Affymetrix’ Expression Console was used to process and normalize raw .cel files using the Robust Multichip Average (RMA) parameters to generate per-gene expression levels. Since the BC and OC cell line experiments were conducted at different time points, data from these cohorts were separately processed and then tested for batch effects. Principle component analysis (PCA) using prcomp in R v3.6.0 revealed the presence of a batch effect between data sets which was corrected using ComBat [38]. After correction, PCA did not reveal any batch effect anymore between the BC and OC cell lines.

Mutational signatures were generated by Jarvis et al. for part of the cell lines (*n* = 53) used in this project [39]. DNA methylation and gene expression data for 50 HR-related genes (listed in Table S3) as well as mutational signature data for base substitution signature 3 for the breast and ovarian cell lines are summarized in Table S4.

### 2.6. BRCA1 Δ11q Splice Variant Detection

cDNA was synthesized previously from total RNA for all BC and OC cell lines as described before [40]. qPCR for the Δ11q splice variant and full-length *BRCA1* was performed in a Mx3000P^TM^ Real-Time PCR System (Agilent, Amsterdam, The Netherlands) using 1X SensiFast SYBR Lo-Rox master mix (Bioline, Memphis, TN, USA) and 333 µM of specific primers (Δ11q-forward 5′-AGAGGCATCCAGAAAAGTATC-3′, FL-forward 5′-GAGCAAAGCATGGATTCAAAC-3′ and reverse 5′-GATAGCCCTGAGCAGTCTTC-3′) as recommended by the manufacturer. In addition to a negative control (i.e., genomic DNA), we also included a standard curve of a serially diluted cDNA sample consisting of pooled BC cell lines in each PCR plate. The latter was performed to ensure that the PCR efficiency between plates was comparable. Presence of the Δ11q splice variant was determined by subtracting the Cq value for the Δ11q splice variant from the Cq value of the full-length *BRCA1* transcript.

### 2.7. Transient Transfection of GFP-SHLD2 cDNA and mScarlet-LMNA CRISPR/Cas9 Assay

HCC1937 cells were grown to 70-80% confluency in a 6-well plate and transfected with 1 µg of the pCW57_1-eGFP-SHLD2 plasmid [17] using transfection reagent Fugene-HD (Thermo Fisher Scientific; DNA:Fugene-HD ratio = 1:4). Twenty-four hours after transfection, 0.5 μg/mL doxycycline was added to the cells to induce *GFP-SHLD2* gene expression. After another 24 h, cells were irradiated and further processed for RAD51 immunofluorescence staining (as described above). pCW57_1-eGFP-SHLD2 was a gift from Sylvie Noordermeer.

HCC1937 and HCC2218 cells were grown to 50–70% confluency in a 6-well plate and transfected with 0.5 and 1 µg, and 1 and 2 µg each of the pReceiver-Lv105-GFP (Genecopoeia, Rockville, MD, USA; EX-EGFP-Lv105), eSpCas9(1.1)_No_FLAG_LMNA_G2 (Addgene, Watertown, MA, USA, #178090) and LMNA_mScarlet-I_Donor (Addgene #178092) plasmids using transfection reagent Fugene-HD (DNA:Fugene-HD ratio = 1:4 and 1:3), respectively. After 72 h, cells were collected and the mean intensity and percentage of GFP- and mScarlet-positive cells were quantified on a FACSAriaIII (BD Biosciences, San Jose, CA, USA). The transfection efficiency was determined by the percentage of GFP-positive cells, whereas the capability of cells to perform HR was determined by comparing the percentage of mScarlet-positive cells with those from a control transfection. This control transfection only lacked the eSpCas9(1.1)_No_FLAG_LMNA_G2, thereby correcting for non-HR-related mScarlet-positivity originating from transfection of the LMNA_mScarlet-I_Donor itself. HRP cell line Hs578T and HRD cell line MDA-MB-436 were used as a positive and negative control to set appropriate gating for the analysis. eSpCas9(1.1)_No_FLAG_LMNA_G2 and LMNA_mScarlet-I_Donor were a gift from Yannick Doyon [41].

### 2.8. Statistical Analyses

Statistical analyses were all two-sided and performed using R v4.2.2 or GraphPad Prism v6.0 (San Diego, CA, USA) for calculating standard errors of the mean in graphs. The Mann–Whitney or Kruskal–Wallis test was used for comparing two or three groups of continuous data, respectively. *p*-values smaller than 0.05 were considered significant.

## 3. Results

### 3.1. Correlation between Functional HR Phenotype and BRCA1/2 Mutations

Since the HR status can change over time, for example, as a result of selective pressure from chemotherapy [42], the prevailing HR phenotype of the 91 BC and OC cell lines was tested using a functional method. This method is based on the formation of RAD51 foci after irradiation, thus reflecting the real-time HR status of the cell line regardless of reported *BRCA1/2* mutation status (Figure S1) [26]. Two of the fifty-three (3.8%) BC cell lines were HRD and one BC cell line was HRI (1.9%; Figure 1). However, ten (18.9%) BC cell lines harbored an inactivating pathogenic *BRCA1/2* mutation. Only two of these (i.e., MDA-MB-436 and HCC1395) were found to be HRD/HRI, while eight *BRCA1/2*-mutant BC cell lines (i.e., HCC1954, SUM149PT, HCC1599, SUM1315MO2, HCC202, HCC1937, HCC1569 and BT474) were functionally HRP. Moreover, HCC2218 was HRD, but wild-type for *BRCA1* and *BRCA2*. None of the thirty-eight OC cell lines were HRD and one (2.6%) was HRI (Figure 2). However, seven (18.4%) OC cell lines harbored an inactivating pathogenic *BRCA1/2* mutation. Only one of these (i.e., PEO1) was also found HRD/HRI, while six *BRCA1/2*-mutated OC cell lines (i.e., UWB1.289+BRCA1, COV362.4, PEO16, COV362, IGROV1 and UWB1.289) were functionally HRP. We thus found a discrepancy between functional HR and *BRCA1/2* mutation status in 15 of the 91 (16.5%) BC and OC cell lines (Table 1).

### 3.2. The HRD Phenotype beyond BRCA1/2 Mutations

**Figure 3:**
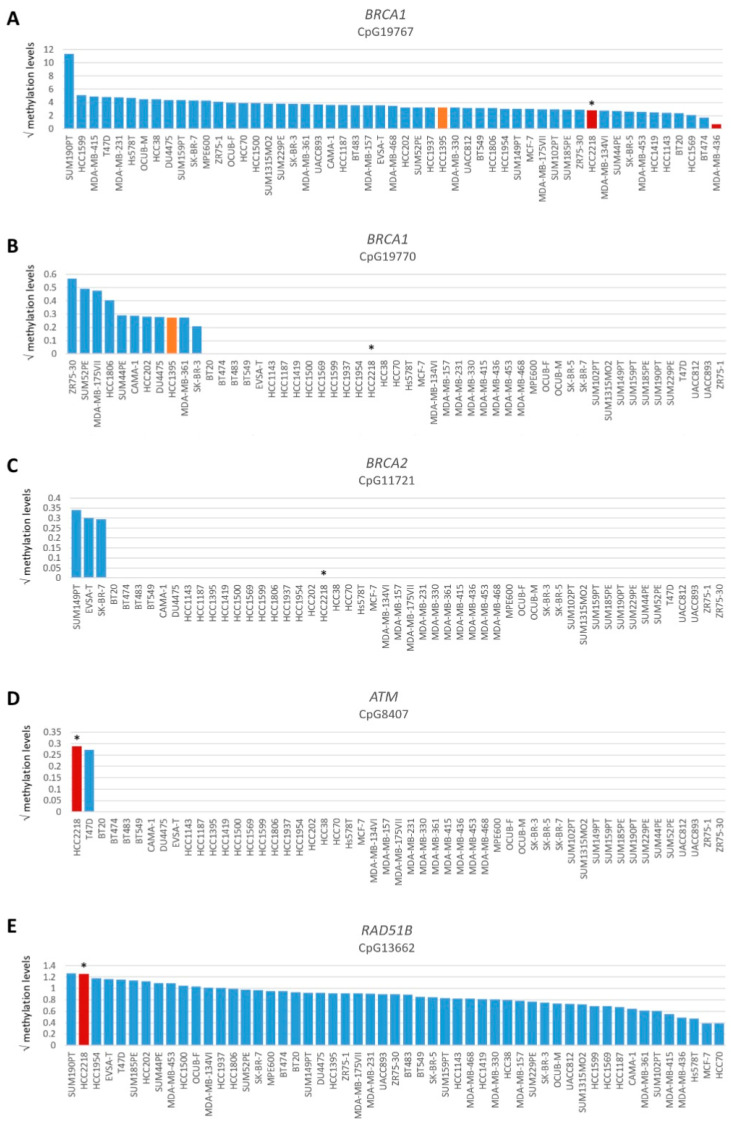
Promoter methylation levels of *BRCA1*, *BRCA2*, *ATM* and *RAD51B*. Graphs present square root-transformed methylation levels of 53 BC cell lines in decreasing order. (**A**) *BRCA1* CpG19767, (**B**) *BRCA1* CpG19770, (**C**) *BRCA2* CpG11721, (**D**) *ATM* CpG8407 and (**E**) *RAD51B* CpG13662. Color of bars illustrates HR status: blue, HRP; orange, HRI and red, HRD. * indicates *BRCA1/2* wild-type HRD cell line HCC2218.

Among all OC and BC cell lines, regardless of *BRCA1/2* mutation status, the incidence of functional HRD was strikingly low. Only two (3.8%) BC cell lines and none of the OC cell lines were HRD. One HRD BC cell line (i.e., HCC2218) was not *BRCA1/2* mutated (Figure 1, Table 1), suggesting alternative means of BRCA1/2 silencing could be the cause of HRD. Therefore, we performed Western blotting of BRCA1, BRCA2 and PARP1 proteins for all HRD/HRI cell lines and BRCA1/2-mutated cell lines regardless of HR status. Cell line HCC2218 showed very low levels of BRCA1 and BRCA2 proteins (Table 1, Figure S2). However, methylation of the *BRCA1* promoter at CpG19767 or CpG19770 or the *BRCA2* promoter at CpG11721 was not evident in this cell line (Figure 3A–C). Therefore, the HRD phenotype of HCC2218 is likely not caused by promoter methylation of either *BRCA1* or *BRCA2*.

Next, we evaluated the exome sequencing data from the 91 BC and OC cell lines for other causes than BRCA1/2 deficiency to explain the HRD phenotype in HCC2218 (Tables S1 and S2). Several other (putative) DNA damage response genes were mutated in HCC2218, such as *ARID1B*, *ATM*, *FANCD2*, *FANCM* and *MRE11*. However, mutations were all missense and heterozygous. Moreover, such mutations were also identified among other BC and OC cell lines not having an HRD phenotype making it unlikely that any of these are causal for the HRD phenotype of HCC2218.

**Figure 4:**
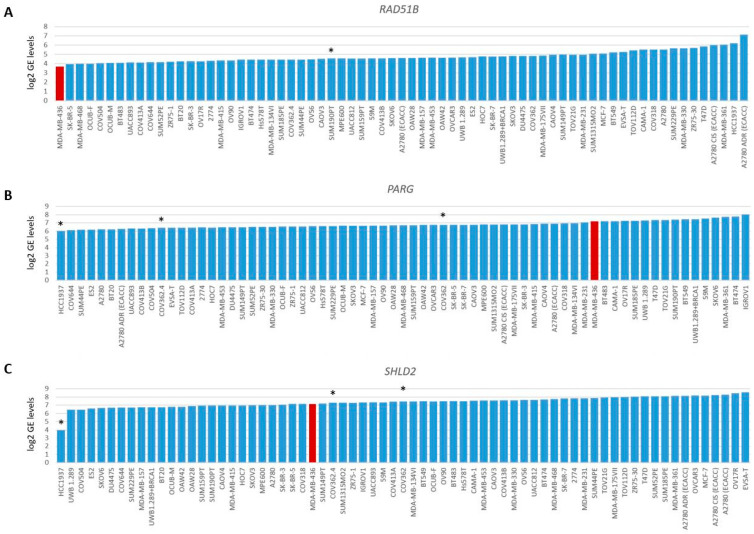
Gene expression levels of *RAD51B*, *PARG* and *SHLD2*. Graphs present log2-transformed gene expression (GE) levels for 69 BC and OC cell lines in increasing order. (**A**) *RAD51B*, * indicates *BRCA1/2* wild-type HRP cell line SUM190PT. (**B**) *PARG* and (**C**) *SHLD2*, * indicates three unexplained BRCA1-deficient HRP cell lines. Color of bars illustrates HR status: blue, HRP and red, HRD.

To evaluate the *BRCA1/2* wildtype HRD phenotype of HCC2218 further, we investigated the promoter methylation status of 60 CpG islands from 50 genes involved in HR (Tables S3 and S4, Figure S3). Interestingly, HCC2218 had the highest promoter methylation levels for *ATM* at CpG8407 and the second highest promoter methylation levels for *RAD51B* at CpG13662 (Figure 3D,E). Unfortunately, we had no gene expression data available for this particular cell line to verify whether promoter methylation of these genes also led to reduced gene expression. However, *ATM* promoter methylation is unlikely to be causal for the HRD phenotype of HCC2218, since pathogenic germline mutations of *ATM* also do not cause HRD [46,47]. Moreover, SUM190PT, the BC cell line with the highest levels of *RAD51B* promoter methylation, did not show reduced but average gene expression levels of *RAD51B*, indicating that *RAD51B* promoter methylation may not always lead to reduced gene expression levels. Thus, promoter methylation of *RAD51B* may also not be causal for the HRD phenotype in HCC2218 (Figure 4A). It thus remains unclear what the underlying mechanism is causing HRD in HCC2218.

### 3.3. Restoration of HR in BRCA1/2-Mutant HRP Cell Lines

Eight BC cell lines (i.e., BT474, HCC202, HCC1569, HCC1599, HCC1937, HCC1954, SUM149PT and SUM1313MO2) and six OC cell lines (i.e., COV362, COV362.4, IGROV1, PEO16, UWB1.289 and UWB1.289+BRCA1) were HRP yet harbored a *BRCA1/2* mutation (Figure 1 and Figure 2). For 6 of these 14 cell lines, the observed HRP phenotype could be explained by a low variant allele frequency (VAF) of the pathogenic *BRCA1/2* mutation (Table 1, Tables S1 and S2). BT474 and HCC1569 harbored a heterozygous *BRCA2* mutation, while *BRCA1/2* mutations in HCC202, HCC1954 and IGROV1 had even lower VAFs (4.3–25%). For HCC1599, the VAF of the *BRCA2* mutation was relatively high (81%), but still resulted in incomplete BRCA2 protein loss. Interestingly, although HCC1569 harbored a heterozygous *BRCA2* mutation and BRCA2 protein levels were low but detectable, BRCA1 protein expression was absent (Table 1, Figure S2). Although we could not find any clear cause for the loss of BRCA1 protein expression among the exome sequencing or methylation data, an HRD instead of the observed HRP phenotype had been expected. Reasons for the HRP phenotype in PEO16 and UWB1.289+BRCA1 are the presence of an in-frame *BRCA2* mutation with corresponding BRCA2 protein expression and reconstitution of BRCA1 function with a wild-type *BRCA1* cDNA, respectively (Table 1 and Table S2). Thus, with the exception of HCC1569, these cell lines with low VAF, an in-frame *BRCA2* mutation and reconstitution of wild-type BRCA1 were all correctly classified as HRP.

PEO1 and PEO4 OC cell lines were also correctly classified. PEO1 and PEO4 were derived from the same patient, but PEO1 was the first platinum-sensitive relapse and PEO4 was a subsequent platinum-resistant relapse. PEO1 harbored a *BRCA2* nonsense mutation that was restored to wild-type in PEO4, rendering PEO4 HRP (Table S2). Moreover, PEO1 was not HRD, but HRI because of a different *BRCA2* restoration mutation than in PEO4. However, this BRCA2 restoration mutation in PEO1 had a VAF of only 30%, resulting in an intermediate HR status (Table 1).

For cell lines HCC1569, HCC1937, SUM149PT, SUM1315MO2 COV362, COV362.4 and UWB1.289, we did not find a *BRCA1/2* gene reversion mutation as the explanation for the HRP phenotype and therefore these seven cell lines are likely to harbor another resistance mechanism that caused restoration of their HR pathway. Indeed, both SUM149PT and UWB1.289 have been shown to express an alternative Δ11q splicing isoform of *BRCA1* [43], whereas SUM1315MO2, on the other hand, expresses a RING domain-deficient BRCA1 protein [44,45]. Both mechanisms lead to partial restoration of BRCA1 proteins and reversion to a HRP phenotype. We confirmed the presence of the alternative Δ11q splicing isoform in our SUM149PT and UWB1.289 cells and additionally identified COV362 and COV362.4 cells expressing this splice variant.

To reveal the underlying cause of the *BRCA1/2*-mutant HRP phenotype of HCC1569 and HCC1937, we evaluated the exome sequencing data for mutations in 13 genes previously shown to be involved in HR restoration, such as *PARG*, *TP53BP1*, *REV7*, *RIF1*, *DYNLL2*, *SHLD* complex genes and *CST* complex genes. For HCC1937, we did not identify mutations in any of these genes, while HCC1569 harbored a missense mutation in *CTC1* as well as two missense mutations in *SHLD3* (Table S1). Missense mutations in these genes were, however, also identified in five BC and OC and two BC cell lines, respectively, and an association with HR reversion was not evident.

Therefore, we also investigated the gene expression data of 48 genes involved in HR. As expected, BRCA1/2-deficient or HRD cell lines did not cluster together upon hierarchical clustering of the expression levels of these genes (Figure S4), since the expression of these genes is linked to cell cycle progression. Next, we searched for outlier behavior (low or high) with regard to the gene expression of the 13 genes previously shown to be involved in HR restoration in *BRCA1/2*-mutant HRP cell line HCC1937. Unfortunately, we had no gene expression data available for HCC1569. Interestingly, HCC1937 had the lowest levels of *PARG* gene expression from the 69 cell lines. However, the low levels of *SHLD2* gene expression were even more pronounced, indicating that reduced *SHLD2* expression could probably be causal for the HRP phenotype in this BRCA1-deficient cell line (Figure 4B,C).

Consequently, we attempted to reconstitute HCC1937 cells with wild-type *GFP*-*SHLD2* cDNA in order to reverse the HRP phenotype. Unfortunately, after transfection of HCC1937 cells with *GFP*-*SHLD2* cDNA and subsequent induction of gene expression with doxycycline, hardly any of the GFP+ cells were in the S-phase of the cell cycle. In total, we observed no more than ten GFP+ cells that were in S-phase and all had low GFP levels, which prohibited us to reliably count RAD51 foci. Since the majority of the ten GFP+ cells, however, seemed to produce RAD51 foci after irradiation (Figure S5), this could suggest that low *SHLD2* expression in HCC1937 cells may not be the cause of the HRP phenotype. However, considering the severely reduced amount of GFP+ cells in S-phase and their low GFP expression, we are reluctant to draw any definitive conclusion.

Finally, to demonstrate that HCC1937 and HCC2218 cell lines were correctly classified as HRP and HRD by the ReCAP assay, we used an HR-dependent CRISPR/Cas9 assay that integrates mScarlet cDNA into the *LMNA* locus [41,48]. For HCC1937 cells, flowcytometry analysis showed an average 2.8-fold increase in mScarlet-positive cells as compared to the control transfection lacking the *LMNA* gRNAs and Cas9 (6.6% vs. 2.3%; Figure S6). In addition, the mean mScarlet intensity was also increased 2.1-fold on average (4867 vs. 2282). For HCC2218 cells, however, the percentage of mScarlet-positive cells and their intensity were comparable to the control transfection (1.55% vs. 1.15% and 1530 vs. 1944; Figure S6). These results confirm that HCC1937 cells are indeed HRP, while HCC2218 cells are HRD.

### 3.4. SBS Signature 3 and Functional HR Status

**Figure 5:**
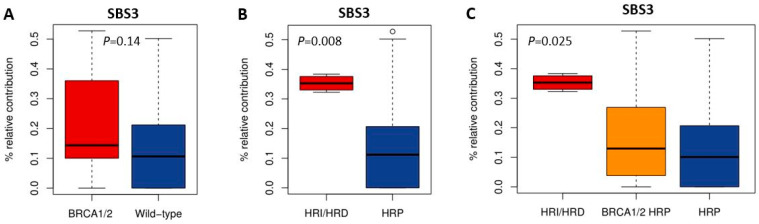
Correlation of SBS signature 3 with *BRCA1/2* mutation and HR status. (**A**–**C**) Boxplots illustrate the difference in median percentage relative contribution of single base substitution signature 3 among the different groups.

HRD-associated mutational scars that can be extracted from massive parallel sequencing data also allow for the classification of HR status. Previously, SBS signatures were extracted from 1020 cancer cell lines by Jarvis et al. [39], also including 37 of the 53 BC and 16 of the 38 OC cell lines studied here. A higher percentage relative contribution (%rc) of SBS signatures 3 and 8 have been associated with BRCA1/2 deficiency and an HRD phenotype. Since only SBS signature 3 could be extracted for these 53 cell lines, we compared classification by SBS signature 3 with *BRCA1/2* mutation status of the cell lines as well as HR status (Figure 1, Figure 2 and Figure 5, Table S4). Interestingly, although *BRCA1/2*-mutant cell lines had a higher median %rc of SBS signature 3 compared with wild-type cell lines, this difference was not statistically significant (*p* = 0.14). In contrast, HRD and HRI cell lines combined did show a significantly higher %rc of SBS signature 3 compared with HRP cell lines (*p* = 0.0080). This was, however, not the case for *BRCA1/2*-mutant HRP compared directly with wild-type HRP cell lines (*p* = 0.67). Importantly, although SBS signature 3 correlated better with functional HR status than *BRCA1/2* mutation status, misclassification of HR status by SBS signature 3 occurred for 6 out the 10 cell lines with a high (i.e., >30%) %rc. Except for the four HRD and HRI cell lines, six HRP cell lines also had a %rc>30%. In fact, the five cell lines with the highest %rc for SBS signature 3 were all HRP. These results highlight the importance of measuring functional HR status instead of *BRCA1/2* mutation status or HRD-associated mutational scars.

## 4. Discussion

In a large cohort of BC (*n* = 53) and OC (*n* = 38) cell lines, we measured the functional HR status by quantifying RAD51 foci formation after irradiation and determined *BRCA1/2* mutation status. Since the HR status can change over time due to evolutionary tumor and treatment selection and cell lines are exposed to genetic drift, we hypothesized that functional HR status better reflects real-time HR status than *BRCA1/2* mutation status. Consistent with this, discrepancies between *BRCA1/2* mutation status and functional HR status were identified in 16.5% (15/91) of BC and OC cell lines. Importantly, while only one of the four (i.e., 25%) HRD and HRI cell lines was not explained by a *BRCA1/2* gene mutation or methylation, the vast majority (i.e., 14/17; 82.4%) of *BRCA1/2*-mutant BC and OC cell lines were found to be HRP. This highlights the importance of determining the functional HR status and additionally could imply a selection advantage for cells to become HRP.

To explain the discrepancies between HR and *BRCA1/2* mutation status for these 15 cell lines, we evaluated exome sequencing, gene expression and promoter methylation data for 50 HR-related genes. Moreover, BRCA1/2 protein expression levels were determined through Western blotting. Despite the amount of relevant data, we were unable to identify a putative causal factor for the HRD phenotype of *BRCA1/2* wild-type BC cell line HCC2218 indicating that novel HRD-causing factors remain to be identified. Alternatively, other mechanisms could be involved such as overexpression of a negative regulator of HR or post-translational inactivation of an HR-related protein [49,50]. Of the fourteen *BRCA1/2*-mutant HRP cell lines, six displayed a low VAF for their *BRCA1* and/or *BRCA2* mutation, one had an in-frame mutation, four had restored BRCA1 function through expression of an alternative *BRCA1* Δ11q splicing isoform, one expressed a RING-less BRCA1 protein [44,45] and one had reconstituted wild-type *BRCA1* cDNA through transfection.

Interestingly, the HRP phenotype of *BRCA2*-mutant BC cell line HCC1569 was explained by its heterozygous *BRCA2* mutation resulting in incomplete BRCA2 inactivation. However, this cell line additionally displayed an absence of BRCA1 protein expression and consequently should have been HRD. We did not find any explanation for the absence of BRCA1 protein or the HRP phenotype of these cells amongst the mutation, methylation or gene expression data. Alternatively, other mechanisms could be involved including alternative splicing, regulation through a microRNA or circular RNA or overexpression of HR proteins that lead to reversion of the HRD phenotype.

Both SUM149PT and UWB1.289 cells had been shown to express a *BRCA1*-Δ11q splice variant lacking the majority of exon 11, thereby partially circumventing both PARPi as well as cisplatin sensitivity arisen from their pathogenic exon 11 *BRCA1* mutations [43]. Similarly, SUM1315MO2 cells had been shown to partially restore BRCA1 function by expressing a RING domain-deficient BRCA1 protein through using an alternative translation start at codon 297. This way, PARPi and platinum-compound sensitivity induced by its RING-domain mutation was reverted, also explaining the HRP phenotype [44,45]. Both BRCA1-specific reversal mechanisms are elegant ways of circumventing RING-domain and exon 11 mutation-induced drug sensitivity. We reconfirmed the Δ11q alternative splice isoform in our SUM149PT and UWB1.289 cells and additionally identified this splice variant in COV362 and COV362.4 cells. We did not, however, observe this variant in IGROV1, which is the only other cell line with a *BRCA1* exon 11 mutation. This is in line with the HRP phenotype of IGROV1 already being explained by a low VAF of its *BRCA1* and *BRCA2* mutations.

Interestingly, COV362 and COV362.4 cell lines both harbored a second *BRCA1* mutation (i.e., c.4096+1G>T) in the splice donor of exon 11. This splicing mutation was unable to revert the upstream c.2612_2613insT mutation, since this insertion’s predicted frame shift (i.e., p.F872Vfs*31) resulted in a premature stop codon far upstream of the c.4096+1G>T mutation. However, it did result in a second transcript being expressed besides Δ11q in which the complete intron 12 was retained and predicted to produce a protein with an in-frame insertion of 134 amino acids.

The *BRCA1*-mutant BC cell line HCC1937 was described as RAD51 foci negative by others, but in our hands, this cell line was able to form RAD51 foci, consequently classifying as HRP [49]. Since cell lines may genetically diverge in time, this is an excellent example of the importance of reconfirming published findings in one’s own cell lines. Importantly, we found a strikingly low *SHLD2* gene expression in HCC1937. SHLD2 is a member of the Shieldin complex, also containing SHLD1, SHLD3 and REV7. The Shieldin complex was shown to promote non-homologous end-joining and the disruption of Shieldin factors restores HR and PARPi resistance in BRCA1-deficient cells [12,13]. However, to our knowledge mutation or loss of *SHLD2* has not been associated with the restoration of PARPi resistance in clinical samples. Therefore, we attempted to establish causality of *SHLD2* downregulation for the HRP phenotype in HCC1937. Perhaps unexpectedly, after reconstitution of *SHLD2* cDNA in HCC1937, the vast majority of SHLD2-expressing cells were not in the S-phase, which is required to assess RAD51 foci formation. In addition, the few cells that were in the S-phase had relatively low SHLD2 expression and were in poor condition. This may suggest that overexpression of SHLD2 is toxic to these cells.

Irrespective of whether *SHLD2* downregulation is causal for the HRP phenotype in HCC1937 cells, 12 of the 14 *BRCA1/2*-mutant HRP cell lines were explained by mechanisms directly acting on BRCA1/2 and not by indirect mechanisms involving PARG, TP53BP1, RIF1, Shieldin or CST complex factors. This is consistent with reports on clinical samples.

We hypothesized that the functional HR status of cell lines would better reflect the real-time HR status of the cells at the time of testing than *BRCA1/2* mutation status. Interestingly, although the %rc of SBS3 was not determined on our own cell lines, but extracted from previously published data, it was significantly higher in HRD and HRI cell lines as compared with HRP cell lines. In contrast, the %rc of SBS3 was not significantly different between *BRCA1/2*-mutant and wild-type cells. There was also no difference in %rc between *BRCA1/2*-mutant HRP and wild-type HRP cell lines. This is an unexpected finding since SBS3-associated mutations induced by BRCA1/2-deficiency would be accumulating in a cell line or tumor during the tumorigenesis process and remain in the genome even after reversal of the HRD phenotype. Therefore, one would expect the %rc of SBS3 to correlate better with *BRCA1/2* mutation status than functional HR status. However, cell lines have been growing ex vivo for a significant amount of time with selection being an ongoing process. Moreover, HRD and HRI cell lines had a significantly higher %rc of SBS3 than *BRCA1/2*-mutant HRP cell lines which could have been accumulated *ex vivo*, after the tumor was removed from the patient. Of course, it is also important to realize that we evaluated only one of five mutational signatures associated with BRCA1/2-deficiency which could have led to some misclassification. In addition, extracting somatic mutational signatures from cell lines without the availability of a matched normal control is much more challenging and signatures were based on whole-exome sequencing data which yields sparser data compared to whole-genome sequence data.

This study provides evidence that BRCA1/2-deficient and/or HRD cell lines comprise a heterogeneous group in which most (i.e., 82.4%) BRCA1/2-deficient cell lines have an HRP phenotype and therefore do not represent HRD tumor specimens. Since cell lines are so broadly applied as a model system, it is of utmost importance to realize that the specific cell line used influences the conclusions that are drawn from these experiments tremendously. Therefore, we recommend not to randomly select a BRCA1/2-deficient cell line, but properly choose the appropriate cell line depending on the purpose of the in vitro experiments. Furthermore, since cell lines including their functional HR status may change over time, the specifics of the cell line should always be verified before commencing a study. Therefore, this comprehensive overview of functional HR status of our collection of cell lines, in addition to previous characterizations, further aids in selecting the proper in vitro model for studies regarding HR, BRCA1/2 function and beyond (e.g., studies utilizing HR-dependent CRISPR/Cas9 gene modification).

## 5. Conclusions

We identified discrepancies between *BRCA1/2* mutation status and functional HR status in 16.5% (15/91) of BC and OC cell lines. Most strikingly, 82.4% (14/17) of *BRCA1/2*-mutant cell lines were HRP, highlighting the importance of determining the functional HR status. Unraveling the mechanisms driving the discrepant HR status in these cell lines demonstrated that reversion mechanisms predominantly acted on restoring the *BRCA1* or *BRCA2* mutation directly. Although we identified one *BRCA1/2*-mutant HRP cell line with a strikingly low *SHLD2* expression, reconstitution experiments were not conclusive in determining causality. Taken together, our results indicate that the majority of *BRCA1/2*-mutant cell lines are not a suitable model to study HRD and thus careful selection of cell lines for in vitro experiments involving HR is warranted. Moreover, only determining *BRCA1/2* mutation status may not suffice for the proper selection of patients for platinum-based chemotherapy or PARPi.

## Figures and Tables

**Table 1:** *BRCA1/2*-mutant and HRD wild-type cell lines.

Cell Line	Pathogenic *BRCA1/2* Mutation	HRD Status	BRCA1/2 Protein	Explanation for Discordance
MDA-MB-436	*BRCA1* c.5396+1G>A, p.E1731del28 and p.I1760ins*8 (VAF = 100%)	HRD	BRCA1 absent, BRCA2 present	HR and BRCA status are concordant
HCC2218	Wild-type	HRD	Very low BRCA1/2 expression	Unexplained
HCC1395	*BRCA1* c.5251C>T, p.R1751* (VAF = 100%) and *BRCA2* c.4777G>T, p.E1593* (VAF = 46.2%)	HRI	BRCA1 absent, BRCA2 present	In-frame *TP53BP1* deletion?
PEO1	*BRCA2* c.4965C>G, p.Y1655* (VAF = 100%) and *BRCA2* c.4964A>T, p.Y1655F (VAF = 29.3%) and *BRCA2* c.682-2A>G (VAF = 10%)	HRI	BRCA1 and BRCA2 present	*BRCA2* c.4964A>T reversion mutation
BT474	*BRCA2* c.9281C>A, p.S3094* (VAF = 46.6%)	HRP	BRCA1 and BRCA2 present	Heterozygous *BRCA2* mutation
HCC202	*BRCA2* c.7033C>T, p.Q2345* (VAF = 4.3%)	HRP	BRCA2 present, low BRCA1 expression	Low VAF *BRCA2* mutation
HCC1569	*BRCA2* c.5578delA, p.V1862* (VAF = 42%)	HRP	Low BRCA2 expression, BRCA1 absent	Heterozygous *BRCA2* mutation
HCC1599	*BRCA2* c.4550_4559del, p.K1517Ifs*23 (VAF = 81%)	HRP	Very low BRCA2 expression, low BRCA1 expression	Incomplete BRCA2 protein loss
HCC1937	*BRCA1* c.5266dupC, p.Q1756Pfs*74 (VAF = 100%)	HRP	BRCA1 absent, BRCA2 present	Low *SHLD2* mRNA expression
HCC1954	*BRCA1* c.5425_5426del, p.V1809Cfs*20 (VAF = 20.6%)	HRP	Low BRCA1 expression, BRCA2 present	Low VAF *BRCA1* mutation
SUM149PT	*BRCA1* c.2169delT, p.P724Lfs*12 (VAF = 96.7%)	HRP	BRCA1 absent, BRCA2 present	BRCA1-Δ11q alternative splice isoform [43]
SUM1315MO2	*BRCA1* c.66_67delAG, p.E23fs*17 (VAF = 100%)	HRP	Low BRCA1 expression, BRCA2 present	RING domain-deficient BRCA1 [44,45]
COV362	*BRCA1* c.2612_2613insT, p.F872Vfs*31 (VAF = 89.6%) and *BRCA1* c.4096+1G>T (VAF = 100%)	HRP	BRCA1 absent, BRCA2 present	BRCA1-Δ11q alternative splice isoform
COV362.4	*BRCA1* c.2612_2613insT, p.F872Vfs*31 (VAF = 94.6%) and *BRCA1* c.4096+1G>T (VAF = 100%)	HRP	BRCA1 absent, BRCA2 present	BRCA1-Δ11q alternative splice isoform
IGROV1	*BRCA1* c.1961delA, p.K654Sfs*47 (VAF = 19.8%) and *BRCA2* c.3320delA, p.K1108Rfs*11 (VAF = 25%)	HRP	Low BRCA1 and BRCA2 expression	Low VAF *BRCA1* and *BRCA2* mutation
PEO16	*BRCA2* c.4141_4143del, p.K1381del (VAF = 94.7%)	HRP	BRCA1 and BRCA2 present	In-frame *BRCA2* mutation
UWB1.289	*BRCA1* c.2475delC, p.D825Efs*21 (VAF = 96%)	HRP	BRCA1 absent, BRCA2 present	BRCA1-Δ11q alternative splice isoform [43]
UWB1.289+BRCA1	*BRCA1* c.2475delC, p.D825Efs*21 (VAF = 68.2%)	HRP	Low BRCA1 expression, BRCA2 present	Reconstitution of wild-type *BRCA1* cDNA

## Data Availability

The data presented in this study are available in the Supplementary Materials.

## Supplementary Materials

The following supporting information can be downloaded at: https://www.mdpi.com/article/10.3390/cancers16040741/s1, Figure S1. Examples of RAD51 staining in breast and ovarian cancer cell lines after irradiation with 5 Gy or without irradiation. Figure S2. Protein expression of BRCA1, BRCA2 and PARP1 in BRCA1/2-mutant or non-*BRCA1/2* HRD/HRI cell lines. Figure S3. Hierarchical clustering of BC cell lines based on the methylation levels of 60 CpG islands from 50 genes involved in homologous recombination. Figure S4. Hierarchical clustering of BC and OC cell lines based on the expression levels of 48 genes involved in homologous recombination. Figure S5. Reconstitution of *GFP-SHLD2* cDNA in HCC1937 BC cells. Figure S6. Verification of the HR status of HCC1937 and HCC2218 with an HR-dependent CRISPR/Cas9 assay targeted to the *LMNA* locus. Table S1. Mutations in 50 HR genes identified by whole-exome sequencing of 53 BC cell lines. Table S2. Mutations in 50 HR genes identified by whole-exome sequencing of 38 OC cell lines. Table S3. List of 50 genes involved in homologous recombination. Table S4. Methylation, gene expression and mutational signature data of breast and ovarian cancer cell lines.
