# Supplementary material for: Functional Homologous Recombination (HR) Screening Shows the Majority of BRCA1/2-Mutant Breast and Ovarian Cancer Cell Lines Are HR-Proficient
Source: Cancers (Basel). 2024 Feb 10;16(4):741. doi: 10.3390/cancers16040741 (PMC10887177; doi:10.3390/cancers16040741)
Supplement: Supplementary file 1 [file cancers-16-00741-s001.zip › RAD51 Cell Lines Figures S1-6.pdf]

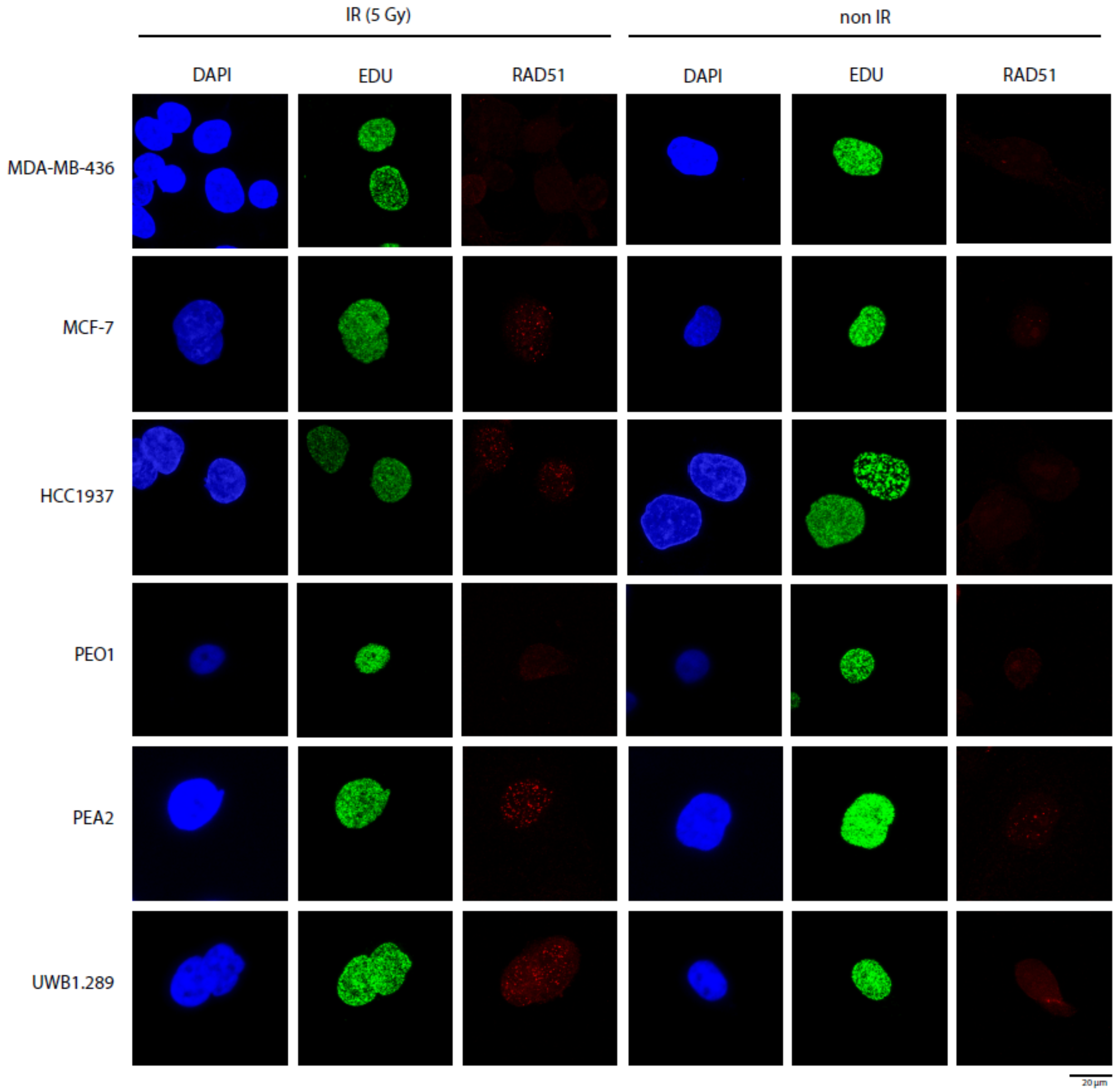

Figure S1. Examples of RAD51 staining in breast and ovarian cancer cell lines after irradiation with 5 Gy or without irradiation. Blue, DAPI; Green, EdU; Red, RAD51.

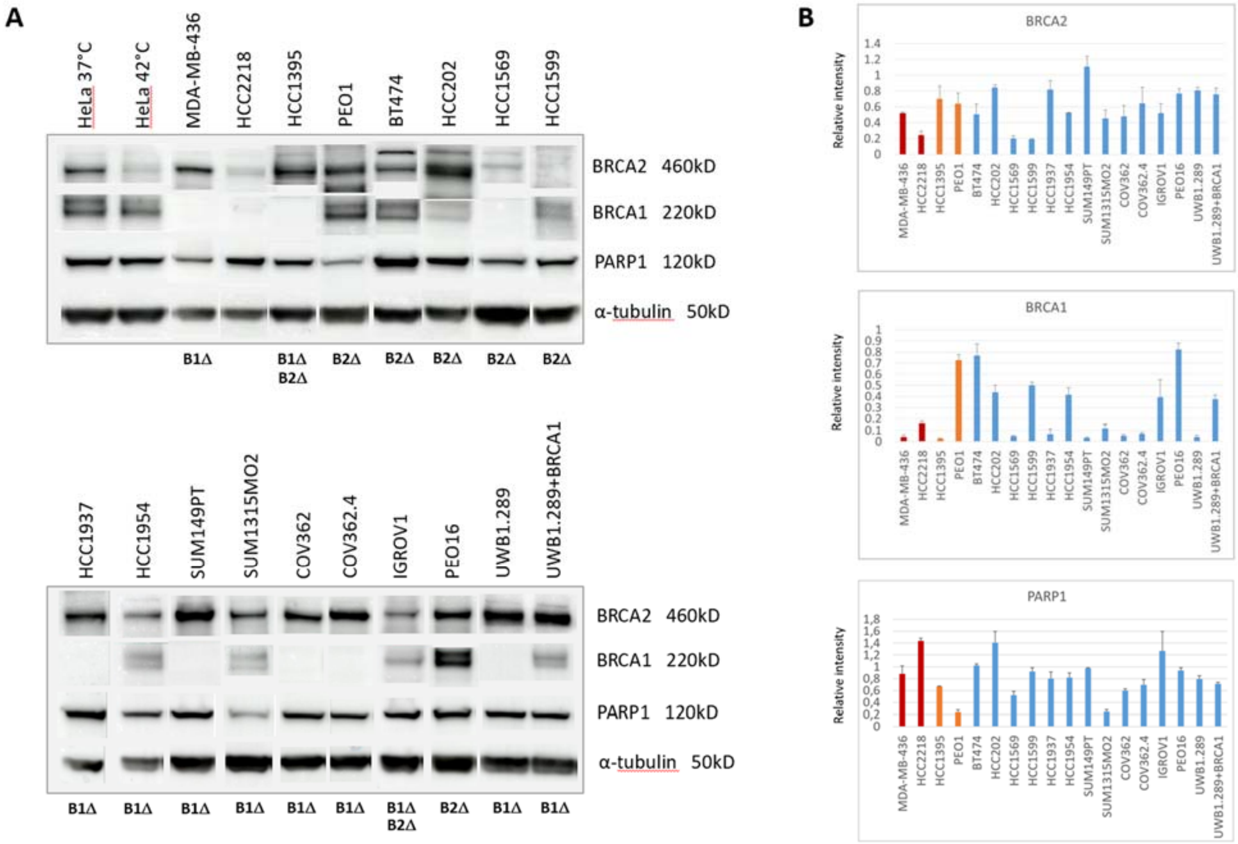

Figure S2. Protein expression of BRCA1, BRCA2 and PARP1 in *BRCA1/2*-mutant or non-*BRCA1/2* HRD/HRI cell lines. A) To determine whether the BRCA2 signal was specific, HeLa cells treated with (42°C) or without hyperthermia (37°C), which causes BRCA2 degradation [47], were included in the experiment. Indeed, the signal designated as BRCA2 was reduced by hyperthermia. α-tubulin was used as a loading control. B1Δ, *BRCA1*-mutant cell line, B2Δ, *BRCA2*-mutant cell line. B) Quantification of BRCA2, BRCA1 and PARP1 western blot bands relative to α-tubulin bands. Each bar represents the mean value of three separate experiments. Error bars indicate SEM. Color of bars illustrates HR status: blue, HRP; orange, HRI; red, HRD.

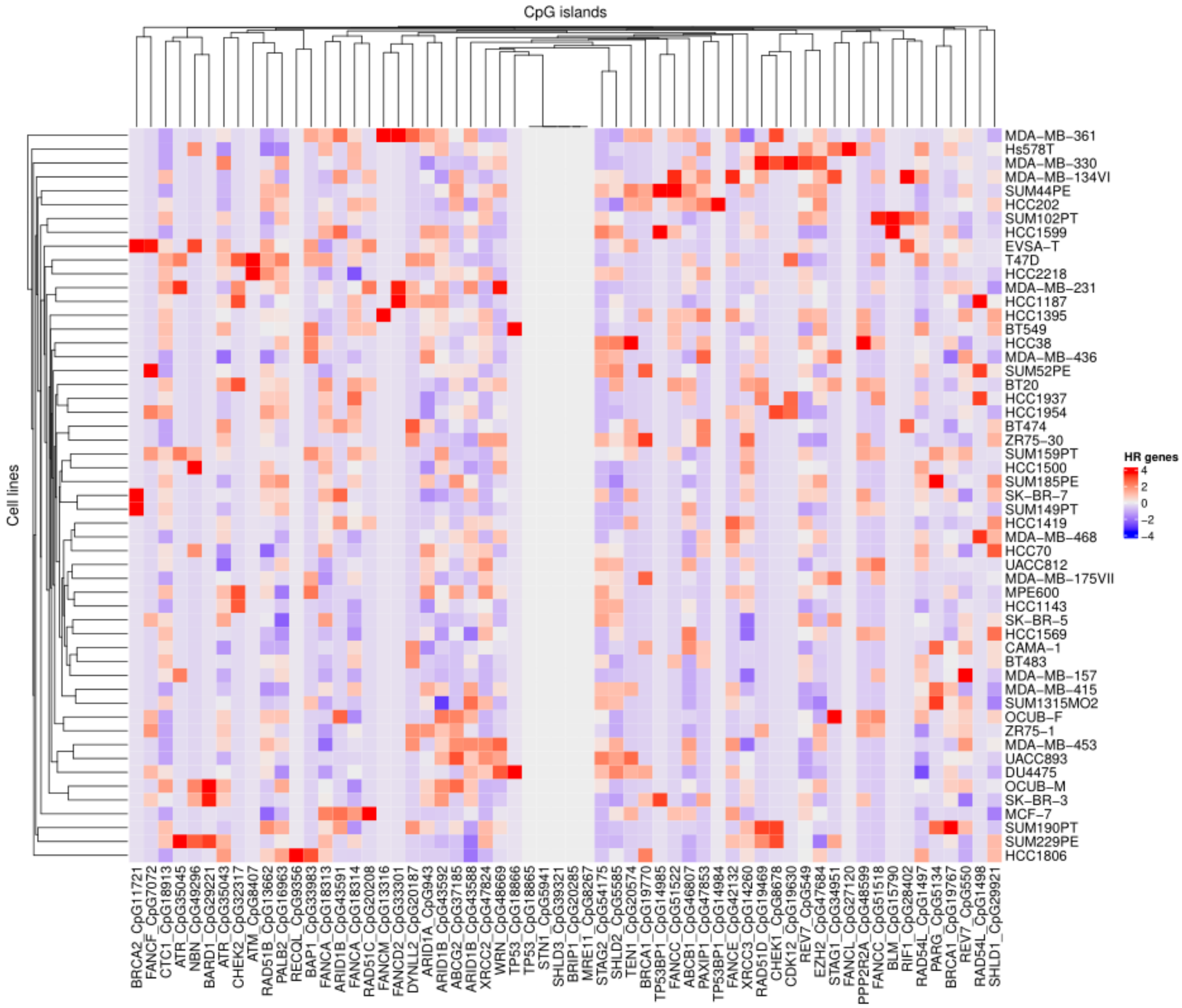

Figure S3. Hierarchical clustering of BC cell lines based on the methylation levels of 60 CpG islands from 50 genes involved in homologous recombination. Supervised clustering was performed using the Euclidean distance metric and average clustering method.

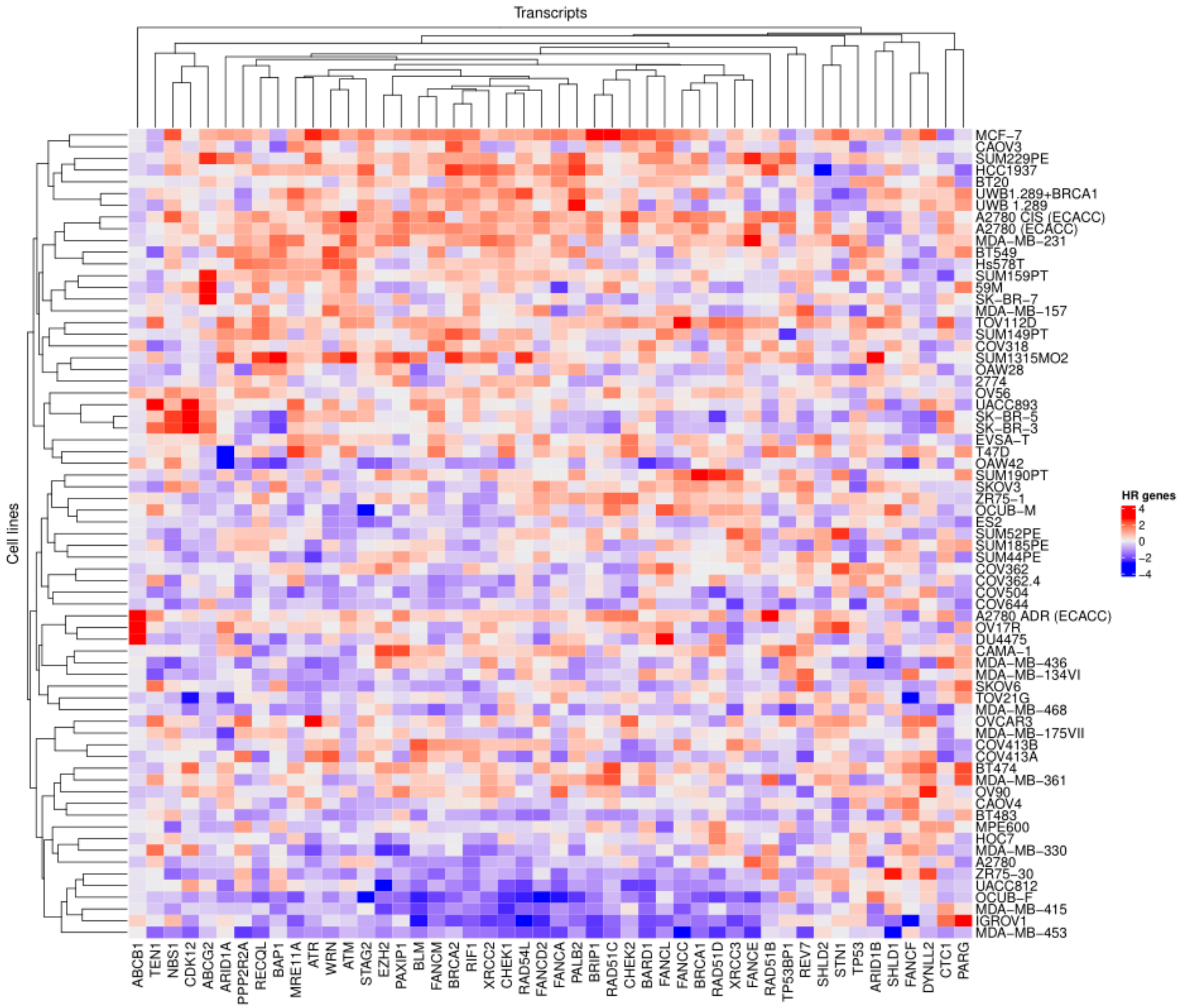

Figure S4. Hierarchical clustering of BC and OC cell lines based on the expression levels of 48 genes involved in homologous recombination. Supervised clustering was performed using the Pearson distance metric and average clustering method.

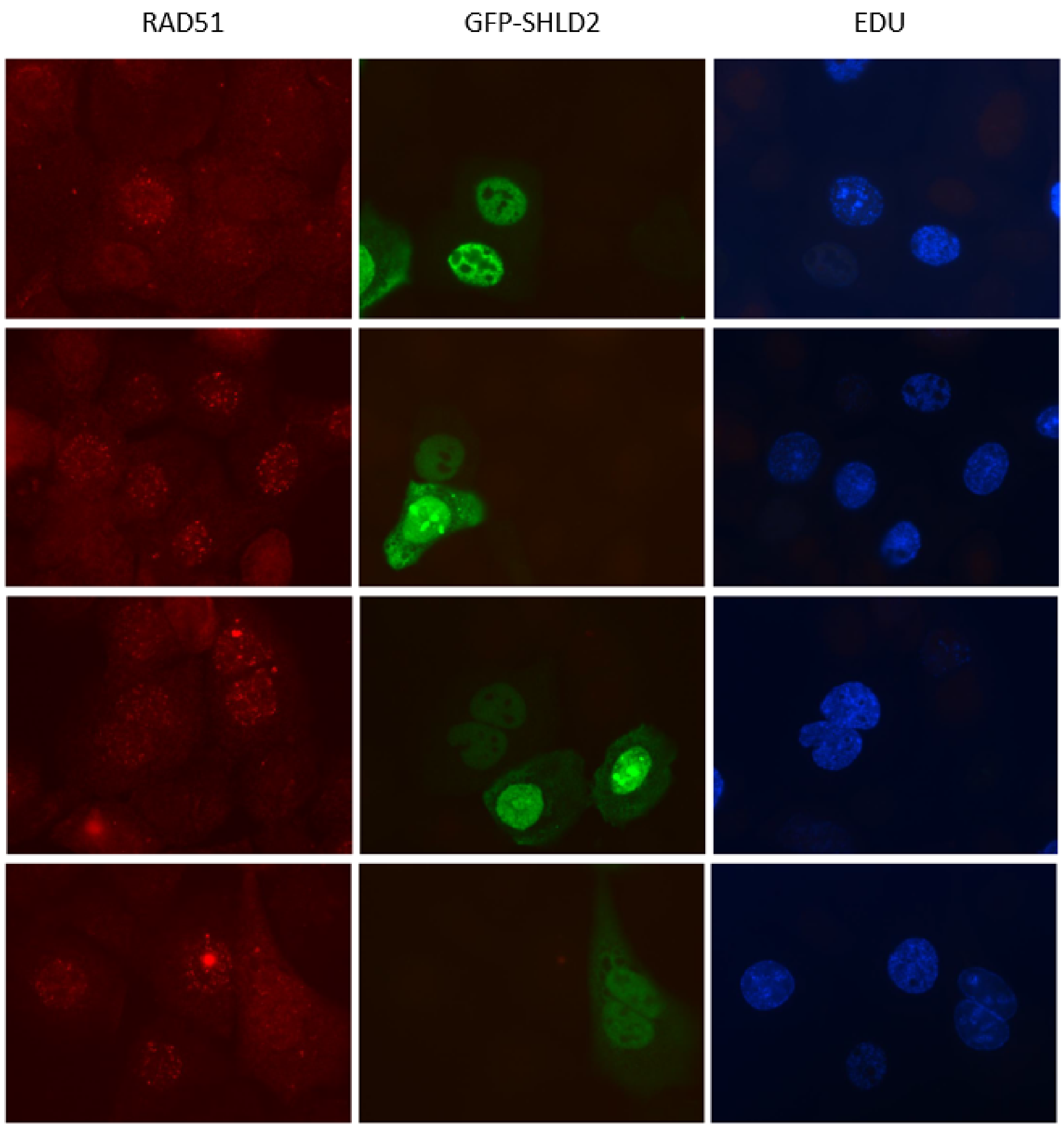

Figure S5. Reconstitution of *GFP-SHLD2* cDNA in HCC1937 BC cells. After reconstitution, cells were irradiated (5 Gy) and stained for RAD51 foci (red) and EdU (blue). The rows represent four different areas with high and low expressing SHLD2-GFP cells.

HCC1937

0.5 μg of each plasmid

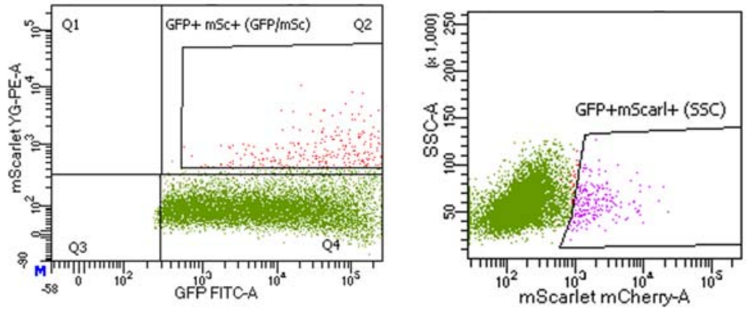

no gRNA/Cas9

Population	#Events	%Parent	%Total
All Events	40,000	####	100.0
cells	28,064	70.2	70.2
FSC single	24,214	86.3	60.5
live (DAPI)	19,172	79.2	47.9
GFP+ (SSC)	9,451	49.3	23.6
GFP+ mSc+ (GFP/mSc)	231	2.4	0.6
GFP+mScarl+ (SSC)	224	2.4	0.6
Q1	0	0.0	0.0
Q2	346	1.8	0.9
Q3	9,630	50.2	24.1
Q4	9,196	48.0	23.0

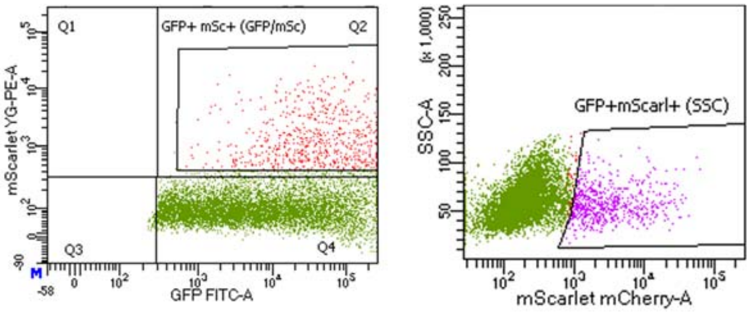

all plasmids

Population	#Events	%Parent	%Total
All Events	40,000	####	100.0
cells	27,808	69.5	69.5
FSC single	23,608	84.9	59.0
live (DAPI)	19,838	84.0	49.6
GFP+ (SSC)	8,885	44.8	22.2
GFP+ mSc+ (GFP/mSc)	662	7.5	1.7
GFP+mScarl+ (SSC)	675	7.6	1.7
Q1	6	0.0	0.0
Q2	869	4.4	2.2
Q3	10,849	54.7	27.1
Q4	8,114	40.9	20.3

1 μg of each plasmid

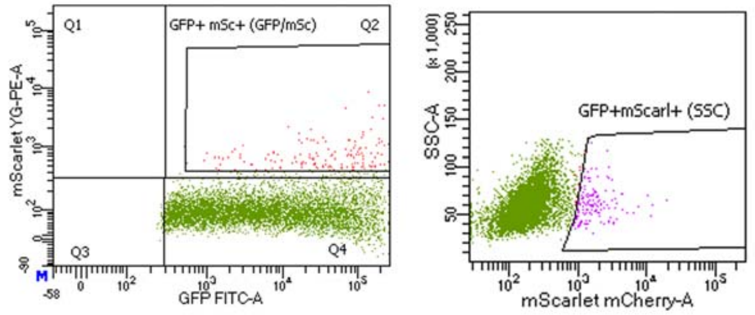

no gRNA/Cas9

Population	#Events	%Parent	%Total
All Events	40,000	####	100.0
cells	27,900	69.8	69.8
FSC single	22,853	81.9	57.1
live (DAPI)	20,477	89.6	51.2
GFP+ (SSC)	6,492	31.7	16.2
GFP+ mSc+ (GFP/mSc)	142	2.2	0.4
GFP+mScarl+ (SSC)	147	2.3	0.4
Q1	8	0.0	0.0
Q2	214	1.0	0.5
Q3	13,909	67.9	34.8
Q4	6,346	31.0	15.9

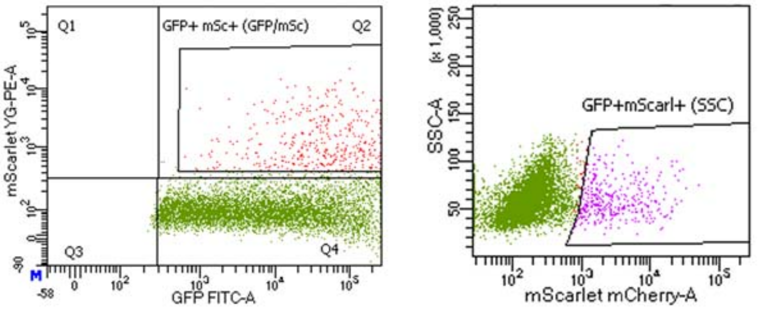

all plasmids

Population	#Events	%Parent	%Total
All Events	40,000	####	100.0
cells	27,758	69.4	69.4
FSC single	23,300	83.9	58.2
live (DAPI)	20,925	89.8	52.3
GFP+ (SSC)	6,894	32.9	17.2
GFP+ mSc+ (GFP/mSc)	387	5.6	1.0
GFP+mScarl+ (SSC)	382	5.5	1.0
Q1	10	0.0	0.0
Q2	479	2.3	1.2
Q3	13,966	66.7	34.9
Q4	6,470	30.9	16.2

HCC2218

1 μg of each plasmid

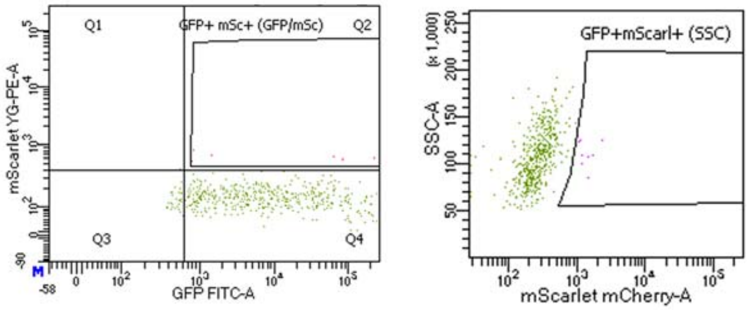

no gRNA/Cas9

Population	#Events	%Parent	%Total
All Events	7,621	####	100.0
cells	7,390	97.0	97.0
FSC single	5,021	67.9	65.9
live (DAPI)	3,629	72.3	47.6
GFP+ (SSC)	467	12.9	6.1
GFP+ mSc+ (GFP/mSc)	6	1.3	0.1
GFP+mScarl+ (SSC)	8	1.7	0.1
Q1	69	1.9	0.9
Q2	19	0.5	0.2
Q3	3,094	85.3	40.6
Q4	447	12.3	5.9

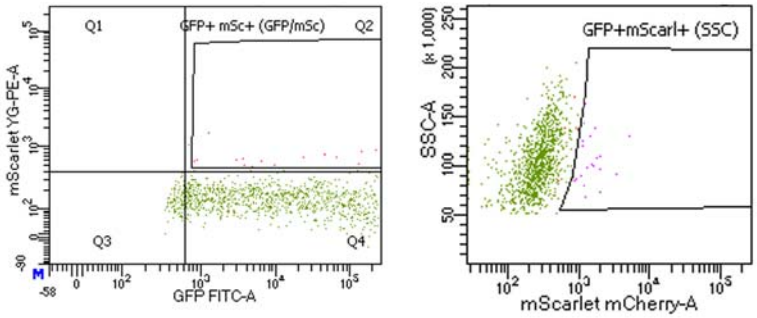

all plasmids

Population	#Events	%Parent	%Total
All Events	14,107	####	100.0
cells	13,668	96.9	96.9
FSC single	9,666	70.7	68.5
live (DAPI)	6,832	70.7	48.4
GFP+ (SSC)	1,044	15.3	7.4
GFP+ mSc+ (GFP/mSc)	17	1.6	0.1
GFP+mScarl+ (SSC)	22	2.1	0.2
Q1	153	2.2	1.1
Q2	69	1.0	0.5
Q3	5,611	82.1	39.8
Q4	999	14.6	7.1

2 μg of each plasmid

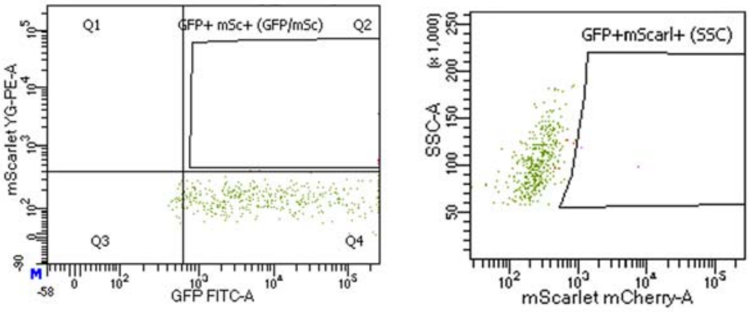

no gRNA/Cas9

Population	#Events	%Parent	%Total
All Events	20,000	####	100.0
cells	19,418	97.1	97.1
FSC single	13,584	70.0	67.9
live (DAPI)	9,430	69.4	47.2
GFP+ (SSC)	419	4.4	2.1
GFP+ mSc+ (GFP/mSc)	4	1.0	0.0
GFP+mScarl+ (SSC)	2	0.5	0.0
Q1	78	0.8	0.4
Q2	6	0.1	0.0
Q3	8,961	95.0	44.8
Q4	385	4.1	1.9

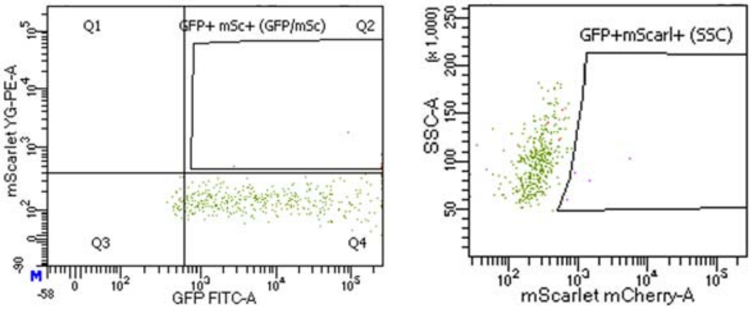

all plasmids

Population	#Events	%Parent	%Total
All Events	18,991	####	100.0
cells	18,414	97.0	97.0
FSC single	12,766	69.3	67.2
live (DAPI)	8,362	65.5	44.0
GFP+ (SSC)	411	4.9	2.2
GFP+ mSc+ (GFP/mSc)	6	1.5	0.0
GFP+mScarl+ (SSC)	4	1.0	0.0
Q1	111	1.3	0.6
Q2	7	0.1	0.0
Q3	7,869	94.1	41.4
Q4	375	4.5	2.0

Figure S6. Verification of the HR status of HCC1937 and HCC2218 with a HR-dependent CRISPR/Cas9 assay targeted to the *LMNA* locus. Cells were transfected with three plasmids containing a GFP cDNA to verify transfection efficiency, two *LMNA* gRNAs and Cas9 cDNA, and a mScarlet-*LMNA* donor sequence.

The control transfection experiment lacked the plasmid containing the mScarlet-*LMNA* donor sequence, thereby correcting for non-HR-related mScarlet-positivity originating from transfection of the donor itself.
